# Contrast-enhanced ultrasound detection of a longitudinal fissure-like intraplaque channel associated with plaque-fragment embolization during carotid artery stenting: a case report

**DOI:** 10.3389/fcvm.2026.1937357

**Published:** 2026-09-14

**Authors:** Wenjing Luo, Renliang Meng, Qingxi Guo, Kai Huang, Jiqing Xuan

**Affiliations:** 1 Department of Ultrasound, The Affiliated Hospital of Southwest Medical University, Luzhou, China; 2 Department of Ultrasound, Jianyang People's Hospital, Jianyang, China; 3 Department of Neurology, The Affiliated Hospital of Southwest Medical University, Luzhou, China; 4 Department of Pathology, The Affiliated Hospital of Southwest Medical University, Luzhou, China; 5 Department of Orthopedics, Jianyang People's Hospital, Jianyang, China

**Keywords:** carotid artery stenting, carotid plaque, case report, contrast-enhanced ultrasound, distal embolization, plaque fissure

## Abstract

**Background:**

Vulnerable carotid plaques with fibrous-cap discontinuity, intraplaque hemorrhage, or an exposed necrotic core may release debris during carotid artery stenting (CAS). Contrast-enhanced ultrasound (CEUS) can dynamically depict plaque-surface abnormalities and intraplaque microbubble perfusion, but a longitudinal, lumen-communicating linear channel within a carotid plaque has rarely been described.

**Case presentation:**

A 75-year-old man presented with 20 days of dizziness, transient syncope, blurred vision, and bilateral lower-limb weakness. B-mode and Doppler ultrasound showed a predominantly hypoechoic plaque with a thread-like, discontinuously perfused residual lumen; spectral Doppler recorded a peak systolic velocity of 367 cm/s. CEUS showed severe stenosis at the origin of the right internal carotid artery and demonstrated microbubbles entering the plaque at its proximal shoulder, traversing a continuous channel parallel to the vessel axis, and returning to the lumen through a distal opening. During CAS, immediately after stent deployment, distal flow abruptly ceased because abundant plaque debris and thrombus obstructed the embolic-protection device. Histopathology of the retrieved material showed cholesterol clefts, fibrinoid necrosis, and intraplaque hemorrhage, confirming that the embolic material contained atherosclerotic plaque rather than thrombus alone. Aspiration restored flow, and no new neurological deficit occurred.

**Conclusion:**

CEUS depiction of a longitudinal, double-opening, lumen-communicating intraplaque channel may provide a preprocedural warning sign of plaque instability and embolic risk during CAS. Recognition of this morphology may support heightened embolic-protection and rescue preparedness, while prospective multimodal studies are warranted to define its reproducibility and clinical significance.

## 1 Introduction

Carotid atherosclerosis is a major cause of ischemic stroke. Plaque composition and morphology influence vulnerability and the risk of distal embolization during carotid artery stenting (CAS) (1). Plaques with a thin or disrupted fibrous cap, a large lipid-rich necrotic core, or intraplaque hemorrhage (IPH) may be particularly susceptible to debris release under the mechanical stress of balloon inflation and stent deployment (2, 3). Plaque fissuring, which differs morphologically from ulceration caused by a full-thickness fibrous-cap rupture, has long been implicated in atherothrombosis (4). Examination of carotid endarterectomy specimens suggests that fissures are not uncommon and are associated with fresh IPH (5). Pathology literature describes a fissure as a small communication between the lumen and necrotic core, often accompanied by IPH and fibrin deposition (6), while prospective imaging data associate IPH with a fissured cap or other plaque-surface disruption (7).

Optical coherence tomography can identify fibrous-cap rupture and fissuring at high spatial resolution, but routine carotid use is limited by its invasive nature and technical requirements (8, 9). Multidetector computed tomography and magnetic resonance imaging can characterize fibrous-cap and plaque-surface morphology, although fine channels may remain difficult to resolve (10–13). CEUS uses microbubbles to enhance blood-flow signals and can dynamically evaluate plaque surfaces, neovascularization, and intraplaque perfusion (14–17). Savastano et al. proposed the concept of intraplaque false-lumen formation; its fenestrated phenotype comprises a channel approximately parallel to the true lumen, with proximal and distal communications (18). This construct provides a morphological reference for the longitudinal, double-opening linear channel seen on CEUS in the present case. However, “longitudinal plaque fissure” is not an established imaging or pathological subtype. We report the CEUS appearance of such a channel and its temporal association with plaque-debris embolization during subsequent CAS.

## 2 Case description

### 2.1 Patient information and clinical findings

A 75-year-old man was admitted after 20 days of recurrent dizziness, transient syncopal episodes lasting 10–20 min, blurred vision, and bilateral lower-limb weakness. Eight years earlier, he had undergone left vertebral artery stenting for recurrent dizziness and related symptoms, with good symptomatic improvement afterward. He had a long smoking history. On admission, he was hemodynamically stable. Neurological examination showed Medical Research Council grade 4 strength in all limbs, without cranial-nerve deficits or pathological reflexes. Total cholesterol (5.28 mmol/L) and low-density lipoprotein cholesterol (3.82 mmol/L) were elevated.

### 2.2 Timeline

‘Timeline’: ‘The timeline of the episode of care is summarized in Table 1’.

**Table 1:** Timeline of the episode of care.

Time	Clinical event
8 years before admission	Left vertebral artery stenting was performed for recurrent dizziness and related symptoms, with subsequent symptomatic improvement.
20 days before admission	Onset of dizziness, bilateral lower-limb weakness, blurred vision, and transient syncope.
Admission	Grade 4 limb strength; lipid abnormalities; carotid imaging performed.
Pre-procedure	CEUS identified severe right internal carotid artery stenosis and a longitudinal, double-opening, lumen-communicating intraplaque channel. Aspirin plus clopidogrel had already been administered for more than 14 days; no clopidogrel loading dose was given.
CAS procedure	CAS was performed through a transfemoral approach with distal embolic protection. Balloon predilation and stent placement were followed immediately by abrupt distal-flow interruption due to plaque debris and thrombus obstructing the protection device.
Immediate rescue	The protection device was retrieved. No dedicated thrombectomy device was used; negative-pressure aspiration was performed through an 8-F guiding catheter, restoring intracranial flow.
Post-procedure	Histopathology demonstrated cholesterol clefts, fibrinoid necrosis, and IPH. CYP2C19 genotyping showed a slow-metabolizer phenotype; clopidogrel was subsequently replaced with ticagrelor.
Postoperative day 7	Discharged with grade 5 limb strength, symptom relief, and no new neurological deficit.

### 2.3 Diagnostic assessment

The patient underwent integrated cervicocerebral vascular ultrasound assessment on a Philips EPIQ 7 system, including extracranial carotid and intracranial arterial evaluation. B-mode ultrasound showed a predominantly hypoechoic, heterogeneous plaque at the origin of the right internal carotid artery (ICA) (Figure 1A). Color Doppler demonstrated thin, discontinuous linear flow signals within the markedly narrowed residual lumen (Supplementary Figure S1A). Spectral Doppler showed markedly accelerated flow, with a peak systolic velocity of 367 cm/s (Supplementary Figure S1B), supporting hemodynamically severe stenosis. Because the lesion had a near-occlusive appearance and the plaque was markedly hypoechoic, the residual lumen could not be delineated reliably on B-mode imaging, and the discontinuous color-flow signal did not permit a reproducible residual-lumen measurement. An exact ultrasound-derived percentage stenosis was therefore not reported. CEUS was performed separately on a Siemens ultrasound platform after intravenous administration of SonoVue (Bracco) and clearly showed a thread-like residual lumen and microbubbles entering the plaque through a luminal opening at the proximal shoulder, coursing through a continuous thin linear channel parallel to the vessel axis, and returning to the lumen through an opening at the distal shoulder (Figure 1B; Supplementary Video). We describe this imaging appearance as a longitudinal, double-opening, lumen-communicating intraplaque linear channel. Its morphology suggested plaque-surface or fibrous-cap discontinuity and resembled fenestrated false-lumen formation.

**Figure 1:**
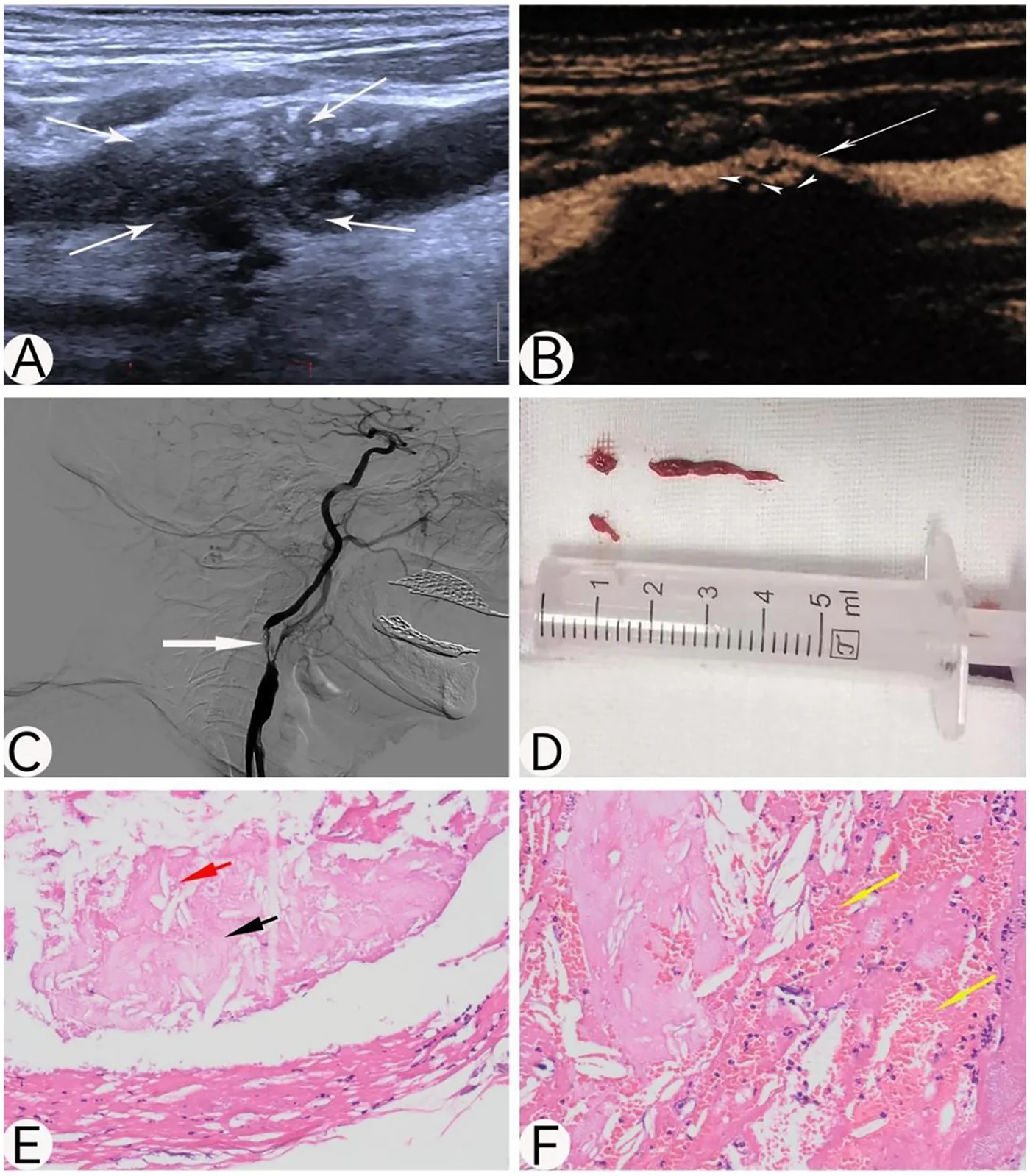
Imaging, retrieved plaque debris, and histopathology. **(A)** B-mode ultrasound shows a predominantly hypoechoic, heterogeneous plaque at the origin of the right internal carotid artery (arrows). **(B)** Contrast-enhanced ultrasound shows the narrow residual lumen (long thin white arrow) and microbubbles entering the plaque at the proximal shoulder, traversing a continuous linear channel parallel to the vessel axis, and returning to the lumen at the distal shoulder (short white arrows). **(C)** Digital subtraction angiography shows severe stenosis at the origin of the right internal carotid artery (white arrow). **(D)** Plaque fragments retrieved from the embolic-protection device. **(E)** High-power histology shows needle-shaped cholesterol clefts (red arrow) and fibrinoid necrosis (black arrow). **(F)** High-power histology shows intraplaque hemorrhage (yellow arrows).

Digital subtraction angiography (DSA) performed at the referring hospital on December 20, 2024, showed linear severe stenosis at the origins of the right ICA and external carotid artery, moderate stenosis of the left ICA origin (approximately 50%) with an ulcerated plaque, and additional multivessel cervicocerebral atherosclerotic disease. The DSA report did not provide a numeric NASCET percentage for the right ICA, and the near-occlusive appearance together with the absence of a reproducible minimum luminal diameter precluded reliable retrospective calculation. CTA was not repeated because DSA had already been obtained at the referring institution and provided the vascular anatomy required for treatment planning. Brain MRI showed a few bilateral frontoparietal and periventricular lacunar/ischemic foci with white-matter changes. These lesions were not hyperintense on diffusion-weighted imaging, no definite acute or recent territorial infarction was documented, and the distribution was bilateral rather than confined to the right ICA territory. Accordingly, the differential diagnosis for the presenting dizziness, syncope, bilateral lower-limb weakness, and blurred vision included vertebrobasilar insufficiency, cardiogenic syncope or arrhythmia, orthostatic hypotension, and other systemic causes.

The case was reviewed by a multidisciplinary team including neurologists, neurointerventional physicians, and a vascular surgeon, with both CEA and CAS considered. Given the patient's diffuse multivessel cervicocerebral disease, the long-segment, very severe right ICA lesion with near-occlusive luminal collapse, and the anticipated complexity of interrupting or adjusting antiplatelet therapy in a patient with a prior vertebral artery stent, open CEA was considered less favorable. CAS was therefore selected as the revascularization strategy. Because the clinical symptoms and MRI findings were not specific to the right ICA territory, the right ICA lesion was treated as a clinically relevant therapeutic target without attributing the presenting symptoms solely to this lesion.

### 2.4 Therapeutic intervention

At the referring institution, aspirin plus clopidogrel had been initiated more than 14 days before CAS; no clopidogrel loading dose was administered. No dedicated platelet-function assay was performed before stenting. CYP2C19 genotyping performed during the hospitalization later showed a slow-metabolizer phenotype. CAS was performed through a transfemoral arterial approach under local anesthesia. An 8-F guiding catheter was positioned in the right common carotid artery, and DSA confirmed the severe right ICA origin lesion (Figure 1C). A SpiderFX distal embolic-protection device (Medtronic/ev3) was deployed. Predilation, which is not routinely performed at our center, was used in this case because of the extreme narrowing; sequential 4 × 30 mm and 5 × 30 mm balloon dilations were performed to facilitate device passage and achieve gradual luminal opening. Representative angiographic images documenting wire/device passage and the final intracranial flow restoration are provided together in Supplementary Figure S2A, while balloon predilation before stent deployment is shown in Supplementary Figure S2B. A 7 × 40 mm Wallstent was selected because the lesion was long and continuous plaque coverage/apposition across the diseased segment was considered desirable.

Immediately after stent deployment, DSA showed a waist in the mid-stent and abrupt interruption of distal flow, consistent with embolic material obstructing the protection device. The device was retrieved with visible plaque fragments and thrombus. Because a filling defect persisted within the stent, the protection device was redeployed and rescue aspiration was performed without a dedicated thrombectomy device. An 8-F guiding catheter (Boston Scientific) was advanced across the stented segment, and negative-pressure aspiration through the guiding catheter removed abundant plaque debris and thrombus. Final angiography documented disappearance of the in-stent filling defect and restoration of distal intracranial flow, including middle cerebral artery perfusion (Supplementary Figure S2A).

### 2.5 Follow-up and outcomes

Histopathological examination of the retrieved material showed needle-shaped cholesterol clefts, fibrinoid necrosis, and IPH, confirming atherosclerotic plaque components and supporting structural instability (Figures 1E,F). Because the sample consisted of fragmented retrieved material rather than an intact endarterectomy specimen, the exact anatomical location of the CEUS-detected channel could not be established. After protection-device retrieval and aspiration, no new neurological deficit occurred. Limb strength improved to grade 5. CYP2C19 genotyping showed a slow-metabolizer phenotype; therefore, clopidogrel was replaced with ticagrelor while aspirin was continued. The patient was discharged on postoperative day 7 with relief of the presenting symptoms. Documented follow-up was limited to the in-hospital period through postoperative day 7; longer-term post-discharge imaging, medication-adherence, and treatment-tolerability data were not available.

### 2.6 Patient perspective

Following treatment, the patient reported that their symptoms had improved compared to before. Although complications arose during the procedure, the patient expressed satisfaction with their overall recovery. After being fully informed about the treatment process and associated risks, the patient indicated that they understood and accepted the treatment decision.

## 3 Discussion

This case documents a clinically coherent sequence: CEUS first demonstrated a longitudinal, double-opening, lumen-communicating intraplaque channel; abundant plaque debris and thrombus then obstructed the distal protection device during CAS; and histopathology confirmed cholesterol clefts, fibrinoid necrosis, and IPH in the retrieved material. Together, these findings support structural plaque instability and a plausible association with mechanically triggered debris release (2, 3, 5–7). Because the specimen consisted of fragmented retrieved material, direct spatial correspondence between the CEUS channel and the retrieved debris could not be established.

The observed CEUS morphology should be distinguished from other plaque-surface and intraplaque findings (Table 2). Plaque fissure generally denotes a small cleft in the fibrous cap or plaque tissue that may communicate with the necrotic core and be associated with IPH and fibrin deposition (5–7). A fissured fibrous cap is also described as an imaging manifestation of plaque-surface disruption (7, 10, 12). Plaque ulceration, by contrast, is a surface recess or crater extending from the arterial lumen into the plaque; a commonly used ultrasound definition classifies a depression with both depth and width ≥2.0 mm as an ulcer (19). Intraplaque neovascularization has a different enhancement pattern: microbubbles are typically seen as punctate or short linear signals arising from the plaque shoulder/adventitial side and extending into the plaque through the vasa vasorum and intraplaque microvascular network, without requiring a direct communication with the arterial lumen (14–16, 19). In the present case, microbubbles entered directly from the arterial lumen through a proximal surface opening, traversed a continuous longitudinal channel, and returned to the lumen distally, favoring a fissure-/false-lumen-type communication rather than neovascular perfusion or a simple ulcer crater. Savastano et al. proposed intraplaque false-lumen formation and plaque excavation as structural phenotypes of plaque disruption; the fenestrated false-lumen phenotype permits blood to enter through a cap defect, traverse a channel, and rejoin the true lumen distally (18). The proximal and distal openings observed here were morphologically closest to this phenotype, providing a plausible structural analogue for the CEUS finding; confirmation of the corresponding pathological substrate would require an intact spatially registered plaque specimen.

**Table 2:** Comparison of the present CEUS finding with related carotid plaque imaging/structural entities.

Entity	Typical structural/imaging appearance	Key distinction from the present CEUS finding	Representative references
Plaque ulceration	Surface recess or crater extending from the lumen into plaque; a commonly used ultrasound criterion is depression depth and width ≥2.0 mm.	Typically a blind-ending surface cavity with a single luminal entrance, rather than a continuous longitudinal channel with distal re-entry.	(10, 11, 19)
Fissured fibrous cap	Small focal cleft or cap discontinuity that may communicate with the necrotic core and is often associated with IPH/fibrin.	Typically focal; a second distal luminal opening is not required.	(5–7, 10, 12)
Fenestrated intraplaque false lumen	Channel approximately parallel to the true lumen with proximal and distal luminal communications.	Closest morphological analogue to the present double-opening longitudinal channel.	(18)
Plaque excavation/necrotic-core cavity	Blood penetrates and excavates a mechanically weak necrotic core after cap disruption, creating a cavity or tract.	May form an intraplaque cavity/tract, but is not specifically defined by a thin CEUS channel with two demonstrated luminal openings.	(18)
Intraplaque neovascularization	Punctate or short-linear microbubble enhancement, often arising from the plaque shoulder/adventitial side and extending inward through the vasa vasorum and intraplaque microvasculature.	Does not require direct luminal entry or a proximal-to-distal re-entry pathway; contrast reaches the plaque through microvascular perfusion rather than a continuous lumen-connected channel.	(14–16, 19)
Present CEUS appearance	Continuous thin channel parallel to the vessel axis with microbubbles entering proximally and returning to the lumen distally.	Operational imaging description; the corresponding pathological substrate remains to be established with an intact spatially registered plaque.	Present case

Treatment-related and antiplatelet factors may also have influenced the intraprocedural event. Protection-device obstruction and plaque-material release are recognized CAS complications (20). Predilation is not routine at our center and was used here because of the extremely severe lesion to facilitate passage and gradual luminal opening; repeated balloon manipulation may nevertheless have contributed to plaque disruption. The abrupt flow arrest occurred immediately after stent deployment. No dedicated platelet-function assay was performed before CAS, and the later CYP2C19 slow-metabolizer result suggests that reduced clopidogrel responsiveness may have contributed to the thrombotic component of the obstruction. Importantly, this does not account for the histologically confirmed plaque fragments. Taken together, the procedural and antiplatelet context supports interpreting the CEUS channel as a preprocedural marker of plaque instability and embolic susceptibility rather than attributing the event to a single mechanism.

The revascularization strategy and stent platform should also be interpreted in an individualized procedural context. Although both CEA and CAS were considered, CAS was selected after multidisciplinary review because of the diffuse multivessel cervicocerebral disease, the long-segment near-occlusive right ICA lesion, and the anticipated complexity of antiplatelet adjustment; this case does not establish superiority of CAS over CEA for plaques with similar high-risk morphology. The 7 × 40 mm Wallstent was chosen to provide continuous coverage and apposition across the long diseased segment. Newer dual-layer or micromesh stent platforms represent alternative plaque-scaffolding strategies, but this single case cannot determine whether a different stent design would have altered the observed plaque-fragment embolization or distal-flow arrest.

Current carotid-plaque risk stratification increasingly integrates stenosis severity with plaque composition and surface morphology. Plaque-RADS provides a standardized multimodality framework for reporting carotid plaque features (21). Ultrasound markers such as echolucency, surface irregularity, juxtaluminal black area, discrete white area, and CEUS-detected intraplaque neovascularization have also been used to characterize plaque vulnerability. In a recent preoperative study, CEUS provided additional information on plaque margins, juxtaluminal black areas, discrete white areas, and neovascularization compared with conventional Doppler ultrasound (22). The finding in the present case differs in that it represents dynamic passage of luminal microbubbles through a continuous longitudinal channel with two apparent luminal communications. If reproduced, this feature may complement rather than replace established imaging markers and standardized risk-stratification systems.

The clinical value of this sign lies in preprocedural risk recognition. When a similar finding is encountered, operators may integrate it with stenosis severity, symptom attribution, plaque composition, antiplatelet status, and procedural complexity; minimize unnecessary balloon manipulation; consider alternative or enhanced embolic-protection strategies where appropriate; and ensure that aspiration equipment is immediately available. These observations support heightened procedural preparedness and provide a basis for future evaluation of protection strategies in patients with similar plaque morphology.

### 3.1 Strengths and limitations

A strength of this report is the multimodal sequence linking dynamic CEUS, procedural angiography, gross retrieval, and histopathological examination. Several limitations should be acknowledged. As a single case, the report cannot define diagnostic accuracy, interobserver agreement, reproducibility, or predictive value. The retrieved material was fragmented, preventing imaging–pathology spatial registration and definitive differentiation among plaque fissuring, excavation of a necrotic core after ulceration, fenestrated false-lumen formation, and other structural channels. CEUS interpretation is operator dependent. The right ICA lesion had a very severe, near-occlusive appearance, but an exact numeric NASCET percentage was not documented in the referring DSA report and could not be reliably reconstructed from conventional ultrasound because the residual lumen was thread-like and poorly delineated. In addition, the symptoms and bilateral chronic-appearing MRI lesions were not specific to the right ICA territory, limiting firm causal attribution. Future studies should prespecify criteria for channel orientation, length, number of openings, and communication with the true lumen; assess interobserver agreement; and seek validation with high-resolution MRI, computed tomography angiography, angioscopy, or intact pathological specimens.

## 4 Conclusion

CEUS visualization of a longitudinal, double-opening, lumen-communicating intraplaque channel before CAS provides a potential preprocedural imaging marker of plaque instability and embolic risk. In this case, plaque-fragment and thrombotic obstruction occurred immediately after stent deployment, and pathology confirmed unstable atherosclerotic plaque components. Recognition of this sign may support heightened attention to embolic protection, procedural manipulation, and rescue preparedness. Prospective multimodal studies are warranted to establish its reproducibility and determine its value within carotid plaque risk stratification.

## Data Availability

The raw data supporting the conclusions of this article will be made available by the authors, without undue reservation.
