# Supplementary material for: Contrast-enhanced ultrasound detection of a longitudinal fissure-like intraplaque channel associated with plaque-fragment embolization during carotid artery stenting: a case report
Source: Front Cardiovasc Med. 2026 Sep 14;13:1937357. doi: 10.3389/fcvm.2026.1937357 (PMC13617083; doi:10.3389/fcvm.2026.1937357)
Supplement: Supplementary file 1 [file Datasheet1.docx]

**Supplementary Figures and Legends**

**Supplementary Figure S1A. Color Doppler imaging of the right internal carotid artery lesion.**

Supplementary Figure S1A. Color Doppler ultrasound acquired during the integrated cervicocerebral vascular examination on a Philips EPIQ 7 system shows thin, discontinuous linear flow signals within the markedly narrowed residual lumen at the origin of the right internal carotid artery. The discontinuous color-flow display is consistent with the near-occlusive morphology and explains why a reproducible residual-lumen diameter could not be obtained from conventional Doppler imaging.

**Supplementary Figure S1B. Spectral Doppler hemodynamic assessment.**

Supplementary Figure S1B. Color-guided spectral Doppler acquired on the same Philips EPIQ 7 system demonstrates markedly accelerated flow across the right internal carotid artery lesion, with a peak systolic velocity of 367 cm/s, supporting hemodynamically severe stenosis. Together, panels S1A and S1B provide the conventional Doppler documentation complementary to the CEUS finding in Figure 1B.

**Supplementary Figure S2A. Angiographic documentation during carotid artery stenting.**

Supplementary Figure S2A. Left: wire/device passage across the severe right internal carotid artery origin lesion during carotid artery stenting. Right: final intracranial digital subtraction angiography obtained after rescue aspiration following stent deployment, demonstrating restoration of distal intracranial arterial flow, including middle cerebral artery perfusion, after the intraprocedural flow interruption.

**Supplementary Figure S2B. Balloon predilation before stent deployment.**

Supplementary Figure S2B. Balloon predilation across the stenotic segment before stent deployment. Predilation, which is not routinely performed at our center, was used in this case because of the extreme narrowing to facilitate device passage and achieve gradual luminal opening.
